# Computational investigation of African natural products as *Helicobacter pylori* shikimate kinase inhibitors

**DOI:** 10.1371/journal.pone.0346899

**Published:** 2026-04-20

**Authors:** Hari Ram Sharma Paudel

**Affiliations:** Department of Chemistry, Tri-Chandra Campus, Tribhuvan University, Kathmandu, Nepal; University of Sahiwal, PAKISTAN

## Abstract

*Helicobacter pylori* is one of the most prevalent bacteria, infecting more than 40% of the world’s population. Rising antibiotic resistance has created an urgent need for the discovery of novel therapeutic agents. One of the potent drug targets for *H. pylori* is shikimic acid pathway, which synthesizes chorismate, an essential precursor for various aromatic compounds necessary for the survival of microorganisms. This pathway is absent in humans but its inhibition is lethal to microorganisms. In this study, shikimate kinase, which catalyzes the fifth step of the shikimic acid pathway, was targeted as a receptor. Potential inhibitors were sourced from the North African Natural Product Database (NANPDB) and the East African Natural Product Database (EANPDB). The natural products were investigated using computational tools. Molecular docking was used to screen the natural products and molecular dynamics simulation was used to assess the dynamic stability of docked protein-ligand complexes. The docking protocol was validated by heavy-atom superimposition of the docked shikimate 3-phosphate onto its co-crystallized conformation. Out of approximately 6500 compounds, six compounds with binding energy ranging from −8.73 to −10.57 kcal/mol were selected from molecular docking. Selected compounds showed stability during 200 ns molecular dynamics simulation. These compounds displayed strong prospects for experimental investigation of possible antibiotics against *H. pylori*.

## 1. Introduction

*Helicobacter pylori* is a gram-negative bacterium that resides in the human stomach. It is estimated that over 40% of the world’s population is infected with *H. pylori* but most people (around 80%) remain asymptomatic [1]. This bacterium mainly causes gastric ulcers which may lead to gastric cancer. *H. pylori* is classified as a class I carcinogen, and in 2022, gastric cancer was ranked fifth in both incidence and mortality worldwide [2–5].

The present therapy of *H. pylori* infection includes a proton pump inhibitor and two broad spectrum antibiotics. But, the resistance to those antibiotics is on rise and hence there is significant decrease in *H. pylori* eradication rate worldwide [6,7]. In addition, extended use of multiple broad spectrum antibiotics causes adverse effects on commensal microbiota and induces dysbiosis [8]. There is an urgent need for developing antibiotics against *H. pylori*.

**Fig 1:**
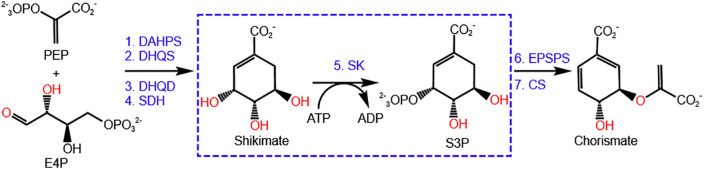
Shikimic acid pathway. The abbreviations are: PEP, phosphoenol pyruvate; E4P, erythrose 4-phosphate; DAHPS, 3-deoxy-D-arabino-heptulosonate 7-phosphate synthase; DHQS, 3-dehydroquinate synthase; DHQD, 3-dehydroquinate dehydratase; SDH, shikimate dehydrogenase; SK, shikimate kinase; S3P, shikimate 3-phosphate; EPSPS, 5-enolpyruvylshikimate 3-phosphate synthase; and CS, chorismate synthase..

The shikimic acid pathway (Fig 1), which synthesizes chorismate from phosphoenol pyruvate (PEP) and erythrose 4-phosphate (E4P) through a series of seven-step enzymatic reactions, is a promising drug target for *H. Pylori* [9,10]. Chorismate is the essential precursor of aromatic compounds including amino acids (phenylalanine, tyrosine, and tryptophan), folate, and vitamins. Shikimate kinase (SK), an enzyme in the fifth step of shikimic acid pathway, catalyzes the stereospecific phosphorylation of the C3 hydroxyl group of shikimic acid by transferring the γ-phosphate group of ATP to the hydroxyl group to provide shikimate 3-phosphate and ADP [11]. This pathway is present in bacteria, fungi and higher plants but absent in humans. The shikimic acid pathway is successfully utilized in synthesizing herbicide. Glyphosate, an herbicide that targets the sixth enzyme (5-enolpyruvylshikimate 3-phosphate synthase) in the shikimic acid pathway, is one of the most widely used herbicides globally; it has been shown to inhibit the growth of apicomplexan parasites *in vitro* [12–14]. Antibiotics developed using this pathway would minimize the possibility of side effects. Thus, this pathway had been an attractive target for antibacterial development due to its absence in humans and lethality of its inhibition.

**Fig 2:**
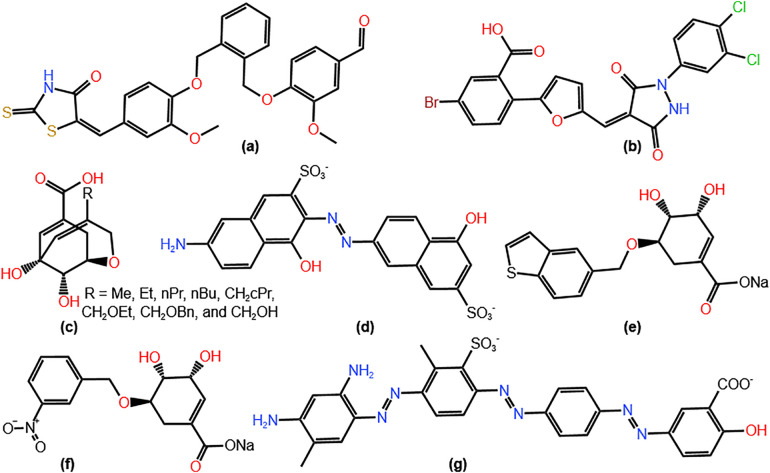
The Structures of known HpSK inhibitors. **(a)** Inhibitor 1, **(b)** Inhibitor 2, **(c)** Inhibitors 3, **(d)** Inhibitor 4, **(e)** Inhibitor 5, **(f)** Inhibitor 6, and **(g)** Inhibitor 7.

Small molecules, including mimetics of shikimic acid derivatives, were synthesized and tested as potential shikimate kinase inhibitors [15,16]. Fig 2 displayed inhibitors of *H. pylori* shikimate kinase (HpSK) reported in literature. Two compounds, inhibitor 1 (IC_50_ = 5.5 **µ**M) and 2 (IC_50_ = 6.4 **µ**M) were discovered by screening the library of 3000 compounds [15]. Shikimic acid derivatives (inhibitors 3) were analyzed against HpSK by computational and inhibition studies. The lowest inhibition constant (K_i_ = 5.0 **µ**M) was obtained for substituent R = CH_2_OEt [16]. Inhibitor 5 (K_i_ = 0.56 **µ**M) and 6 (K_i_ = 0.46 **µ**M), also derivatives of shikimic acid, showed an inhibitory effect against HpSK [17]. Inhibitor 4 (IC_50_ = 4.9 **µ**M) was used to solve the crystal structure of Lys114A HpSK variant enzyme (PDB ID: 3N2E). It prevented the closure of the active site for catalysis [18,19]. Likewise, inhibitor 7 (IC_50_ = 4.8 **µ**M) was one of the six inhibitors of HpSK identified from the NCI database [20]. Seven HpSK inhibitors were identified by computational screening of 1615 FDA-approved agents [21]. In addition, natural products from plants were investigated computationally and experimentally as potential antibacterials against *Bacillus subtilis* shikimate kinase and *H. pylori* urease inhibitors [22,23]. The literature survey revealed that while the shikimic acid pathway is a promising drug target, the number of inhibitors exploiting HpSK is limited.

Traditionally, people in different parts of the world have used phytochemicals from herbs as medicine for stomach related problems including gastric ulcers. Various types of herbs and extracts are in practice even today. In addition to plant-derived natural products, marine natural products also have shown potential to be a source of drug molecules [24]. Natural products could serve as effective antibiotics against *H. pylori*. Here, natural products from the North African Natural Product Database (NANPDB) and the East African Natural Product Database (EANPDB) (approximately 6500 compounds) are selected as possible drugs against *H. Pylori*. A receptor protein: *H. pylori* shikimate kinase (HpSK) from the shikimic acid pathway is selected as target.

### 1.1. The structure of *H. pylori* shikimate kinase

**Fig 3:**
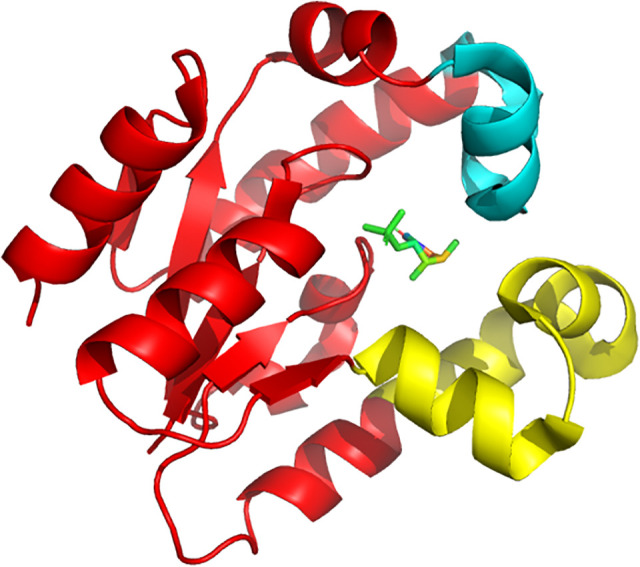
HpSK (PDB ID: 3MUF) with three regions. core (yellow), SB (magenta), and lid (cyan) with shikimate 3-phosphate (S3P) in the binding pocket.

Shikimate kinase has a recognition center for shikimic acid and its cofactor ATP [19]. The HpSK consists of three domains (Fig 3): the CORE domain (residues 1–31, 61–108, and 124–162) that contains five stranded parallel β-sheets and the P-loop, which forms the binding site for ATP and ADP; the substrate binding (SB) domain (residues 32–60), which is responsible for the recognition and binding of shikimic acid; and the LID region (residues 109–123), which has an essential arginine for the binding of ATP [11,18,25,26]. The LID region is most flexible and covers the binding pocket which allows in and out of substances.

### 1.2. Binding site of HpSK

Structural and computational investigations have identified the key residues responsible for binding within the HpSK active site. The mechanism of catalysis of shikimate kinase revealed that ATP first binds to the enzyme and induces a large movement of the LID domain over the active site. Shikimic acid subsequently binds to the active site and the LID domain closes over the active site [27]. According to a two step mechanism from quantum mechanical and molecular mechanical (QM/MM) methods, residues Lys14, Arg1116, and Asp33 are involved in transfer of phosphate from ATP to C3 position of shikimic acid [28]. Structural study from HpSK has identified that residues Asp33, Arg57, Arg116, Arg132, Gly79, Gly80, Gly81, Glu114, Met10, Val44, and Phe48 bind with the shikimate. But, site-directed mutagenesis study on those shikimate binding residues (Asp33, Arg57, Arg116, Arg132, Met10, Phe48, and Glu114) revealed that three conserved Arg residues (Arg57, Arg116, and Arg132), the side chain of Asp33, and the aromatic ring of Phe48 are involved in binding to shikimate [18,25].

## 2. Materials and methods

### 2.1. Ligands and receptor preparation

Ligands were downloaded from the North African Natural Product Database (NANPDB) and the East African Natural Product Database (EANPDB), which were available at https://african-compounds.org [29,30]. Macrocycles (ring size ≥ 7) were excluded from the docking study because they require a specialized docking protocol [31]. They were filtered using RDKit software. The selected compounds were optimized with openbabel [32] using universal force field. Ligands were converted into PDBQT format using the Meeko tool from the Forli lab at the Center for Computational Structural Biology (CCSB, https://github.com/forlilab/Meeko, accessed on April 1, 2025).

Crystal structures of receptor *Helicobacter Pylori* Shikimate kinase (HpSK, PDB ID: 3MUF) was downloaded from Protein Data Bank (PDB, http:/www.rcsb.org/). Heteroatoms and water molecules were removed from the structure. Subsequently, polar hydrogens were added to the receptor using Open-Source Pymol [33]. The receptor was converted to PDBQT format using Meeko tool.

### 2.2. Molecular docking

Molecular docking of the selected compounds with HpSK (PDB ID: 3MUF) was carried out by using Autodock Vina version 1.2.5 [34]^.^. The binding pocket of receptor protein was identified through literature searches and the use of the ADFR software suite [35]. Grid box center (Å) was set at: X = 27.19, Y = 29.60, and Z = −1.50. Grid box dimensions (Å) were set as X: 24.00, Y: 22.00, and Z: 25.00. The top six compounds displaying good binding energy, hydrogen bonding, and noncovalent interactions were selected. The selected compounds were re-optimized with mmff94 force field using openbabel and converted to PDBQT format using Meeko tool. Then, the compounds were re-docked using Auto Dock Vina with exhaustiveness: 32, and maximum number of evaluations: 10 million. Inhibition constant (K_i_, µM), the dissociation constant of reversible enzyme-inhibitor complex (EI, Equation 1), was calculated by using their binding energies (ΔG, kcal mol^-1^) according to the Equations 2 and 3:


$$E+I\begin{array}{c} K_{eq}\\ \to \\ \leftarrow \\ K_{i} \end{array}EI$$ (1)

$$\Delta G=-RT\text{ln}K_{eq}$$ (2)

$$K_{i}=\frac{\text{1}}{K_{eq}}$$ (3)

where, R is the universal gas constant (1.987[CTRL U+008E]10^−3^ kcal mol^-1^ K^-1^), T is the temperature (298.15 K), E is the enzyme, I is the inhibitor, and K_eq_ is the equilibrium constant for the formation of enzyme-inhibitor complex [36–39]. The smaller value of K_i_ indicates lower dissociation probability for docked protein-ligand complex and hence higher inhibition capacity of ligand [40]. The interaction models of protein-ligand complexes were produced using the Biovia Discovery Studio 2021 [41].

### 2.3. Molecular dynamics simulations

Molecular dynamics (MD) simulation was performed using Gromacs [42] version 2024. The best pose of ligands from docking was extracted and hydrogens were added using ChimeraX [43] software. Then, the bond orders of ligands were fixed with Avogadro [44]. Ligand topologies were generated with AnteChamber PYthon Parser interfacE (ACPYPE) using General Amber Force Field (GAFF2) [45]. Protein topology was generated with pdb2gmx tool of Gromacs using the Amber ff99SB-ILDN force field [46]. A cubic simulation cell was created and three site transferable intermolecular potential (TIP3P) water system was added. Sodium and chloride ions were used to neutralize the system. The energy minimization was performed with the steepest descent algorithm with maximum force 1000 kJ mol^-1^ nm^-1^. Each protein-ligand complex was equilibrated for 1000 ps at constant number of molecules, volume, and temperature (NVT) ensemble, followed by an MD run of 1000 ps in a constant number of molecules, pressure, and temperature (NPT) ensemble, with positional restraints applied on protein heavy atoms. Then, production runs of 200 ns were conducted with NPT ensemble without positional restraints. The temperature was maintained at 300 K by stochastic velocity rescaling [47], and pressure was maintained at 1 bar by a Parrinello-Rahman barostat [48]. The temperature and pressure time constants were set to 0.1 ps and 2 ps, respectively. Covalent bonds involving hydrogen atoms were constrained using the LINC algorithm [49]. The time step of MD simulation was set at 2 fs and MD trajectories were saved at 5000 steps (10 ps) intervals. The conformational stability of the protein-ligand complexes were assessed by the the root mean square deviation (RMSD) of the protein backbone atoms and ligand heavy atoms from the production run with respect to the structure configuration of protein backbone atoms after the equilibration run. The root mean square fluctuation (RMSF) of protein backbone atoms, radius of gyration (Rg), and hydrogen bonds were analyzed for the MD simulation production runs.

## 3. Results and discussion

### 3.1. Molecular docking study of HpSK

**Fig 4:**
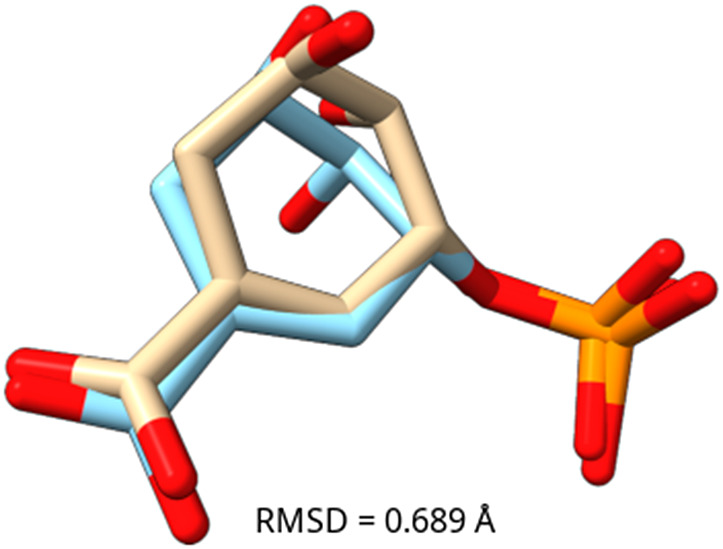
Superimposition of docked (pale yellow) and co-crystallized (sky blue) ligand pose of S3P. The RMSD observed was 0.689 Å.

The co-crystallized ligand, shikimate 3-phosphate (S3P), in the crystal structure of HpSK (PDB ID: 3MUF), was used as the reference for docking studies. The molecular docking was validated by superimposing the docked S3P onto its co-crystallized pose, using only heavy atoms. The superimposition (Fig 4) yielded a root-mean-square deviation (RMSD) of 0.689 Å, confirming the docking protocol’s accuracy (RMSD < 2.0 Å) [50,51]. Table 1 showed the overview of interaction of selected compounds to HpSK. Compound 1 had the highest binding energy of −10.57 kcal/mol among all the compounds. The binding energy for S3P (reference) was −8.01 kcal/mol whereas all other selected compounds had binding energy greater than that of the S3P. Computed Inhibition constants of the compounds are also listed.

**Table 1:** Binding energy, inhibition constant, hydrogen bonding, and other noncovalent interactions of compounds.

Cpds	Binding Energy (kcal/mol)	Inhibition Constant (µM)	Hydrogen Bonding Interaction				Other Non-covalent Interactions		
1	−10.57	0.018	Arg57	Thr78	Ser77	Ser15	Asp33	Arg116	Lys115
							Met10	Gly80^*^	Val44^†^
2	−9.95	0.051	Gly11	Ser12	Gly13	Lys14	Arg116	Asp33	Met10
			Ser15				Pro117	Lys14	Val44
3	−9.77	0.069	Arg116^2^	Lys14	Ser15		Arg116	Asp33	Val44
							Gly80^*^	Met10	Thr78
4	−9.52	0.105	Arg116	Thr78	Ser12	Ser15	Lys115^†^	Met10	Lys14
			Arg57	Asp31	Gly11	Gly81	Arg57^*^	Val44	Arg116
			Lys14				Asp31		
5	−9.06	0.227	Arg57	Arg132	Asp33	Asp31	Arg116	Lys14	Ser15
			Glu60	Gly11	Ser12	Gly13			
			Lys14^2^						
6	−8.73	0.398	Gly13	Glu112			Arg116	Val44	Met10
							Asp33	Ser15	Lys115
							Gly13^*^	Gly11^*^	
S3P.	−8.01	1.345	Arg116	Arg132	Arg57^2^	Gly80^2^	Met10	Lys14	Asp33^*^
			Asp33^2^	Lys14			Arg57	Gly79	Arg116
							Pro117	Arg132	

* indicates unfavorable interaction, † indicates favorable and unfavorable interaction, and the number in superscript indicates number of interactions.

**Fig 5:**
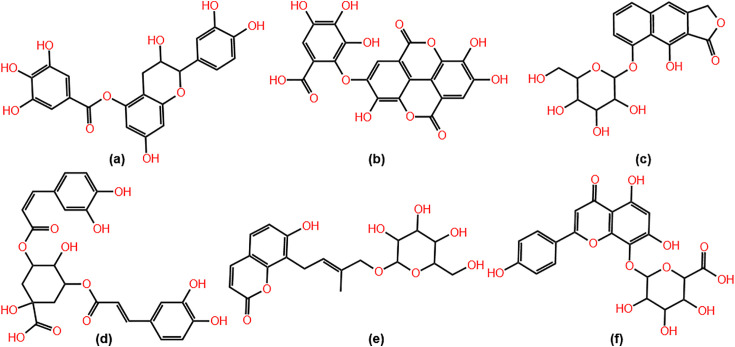
Structures of selected compounds. **(a)** Compound 1, **(b)** Compound 2, **(c)** Compound 3, **(d)** Compound 4, **(e)** Compound 5, and **(f)** Compound 6. Structures were generated with KingDraw software.

**Fig 6:**
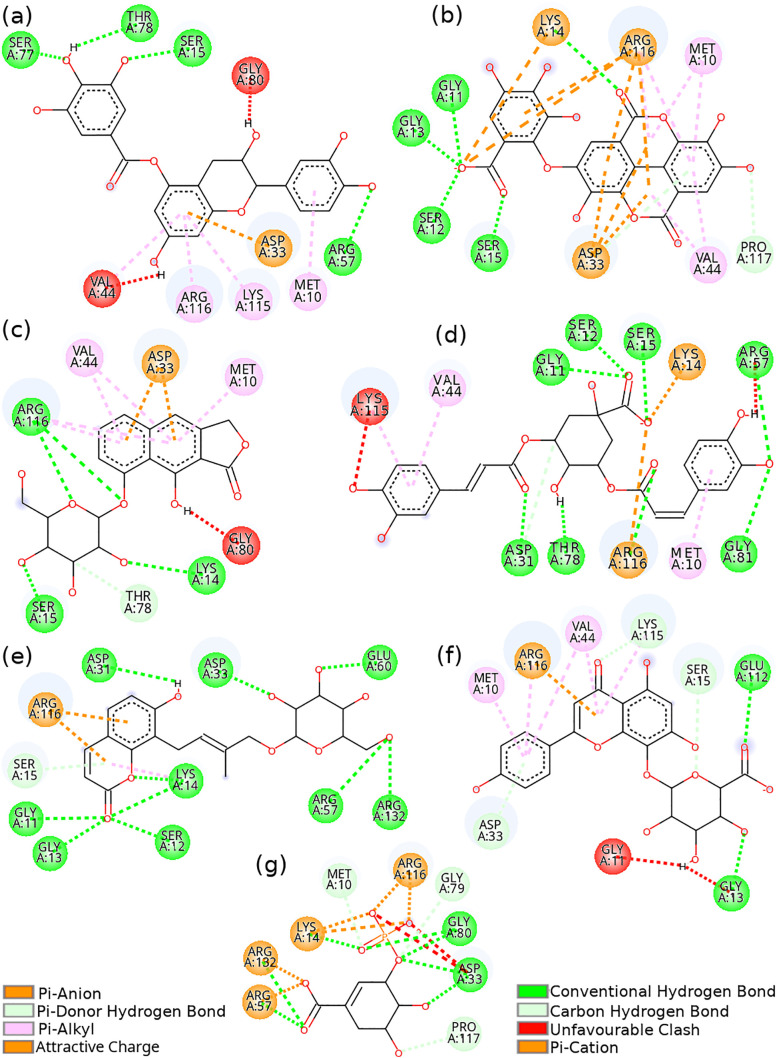
Interaction diagrams of HpSk with selected compounds. **(a)** HpSK − cpd 1, **(b)** HpSK − cpd 2, **(c)** HpSK − cpd 3, **(d)** HpSK − cpd 4, **(e)** HpSK − cpd 5, **(f)** HpSK − cpd 6, and **(g)** HpSK − S3P. Key amino acids contributing to interactions are shown in circles; Compound is represented in line model. The light blue halo surrounding the interacting residues represents the solvent-accessible surface. Implicit and non-interacting hydrogens are not shown. Unfavorable clash means unfavorable donor-donor, acceptor-acceptor, or negative-negative interaction..

The structures of selected compounds 1–6 are displayed in Fig 5 and their docked complexes with HpSK are shown in Fig 6. Compound 1 formed hydrogen bonds with residues Ser77 (d = 2.32 Å), Thr78 (d = 1.92 Å), Ser15 (d = 1.98 Å), and Arg57 (d = 2.01 Å). All the hydrogen bonds in compound 1 were short hydrogen bonds (d < 2.7 Å) [52] which contributed to its highest binding energy. Other noncovalent interactions of compound 1 included pi-alkyl (Met10, Arg116, Lys115, and Val44) and pi-anion (Asp33) interactions. However, its binding was compromised by unfavorable donor-donor interactions with residues Val44 and Gly80.

Compound 2 showed hydrogen bonding with Lys14 (d = 2.02 Å), Gly11 (d = 2.20 Å), Ser12 (d = 2.65 Å), Gly13 (d = 2.49 Å), and Ser15 (d = 2.16 Å). In compound 2, Asp33 formed three pi-anion interactions and one pi-donor hydrogen bond. Likewise, Arg116 formed three pi-cation interactions, one attractive charge interaction, and three pi-alkyl interactions. Here, the Asp-pi-Arg interaction was stabilized by cooperativity of anion-pi-cation ternary type interaction [53]. Other interactions of compound 2 were with residues Met10 (two pi-alkyls), Lys14 (attractive charge), Pro117 (carbon hydrogen bond), and Val44 (two pi-alkyls). Residues Arg116, Asp33, val44, and Met10 from binding site had interaction with compound 2. Hydrogen bond of Lys14 was assisted by aromaticity modulation of the cyclic ring containing carbonyl group, which in turn assisted to stabilize pi-alky interaction of the ring [54,55]. Compound 3 was stabilized by hydrogen bonds of residues Ser15 (d = 2.44 Å), Lys14 (d = 2.29 Å), and Arg116 (d_1_ = 2.30 Å and d_2_ = 2.88 Å). In addition, it had pi-anion (Asp33), pi-alkyl (Arg116, Val44, and Met10), carbon hydrogen bond (Thr78), and donor-donor clash (Gly80) interactions. Compound 3 had two intramolecular hydrogen bonds between hydroxyl groups of glycosidic ring, which is believed to increase permeability of compound at the expense of solubility [56,57].

Compound 4 formed hydrogen bonds with residues Arg57 (d = 2.35 Å), Arg116 (d = 2.18 Å), Ser12 (d = 2.85 Å), Lys14 (d = 2.15 Å), Ser15 (d = 2.14 Å), Thr78 (d = 2.46 Å), Gly11 (d = 2.19 Å), Gly81 (d = 2.18 Å), and Asp31 (2.09 Å). Other interactions included carbon hydrogen bond (Asp31), pi-alkyls (Met10, Val44, and Lys115), and attractive charge-charge interactions (Lys14 and Arg116). Compound 4 had unfavorable acceptor-acceptor and donor-donor interactions with residues Arg57 and Arg45, respectively. Compound 5 formed hydrogen bonds with Arg57 (d = 2.49 Å), Arg132 (d = 2.18 Å), Lys14 (d_1_ = 2.35 Å and d_2_ = 2.37 Å), Ser12 (d = 2.52 Å), Gly13 (d = 2.17 Å), Gly11 (d = 2.51 Å), Glu60 (d = 2.94 Å), Asp33 (d = 2.01 Å), and Asp31 (d = 2.85 Å). Interactions other than hydrogen bonds included pi-cation interactions of Arg116, pi-donor hydrogen bond of Ser15, and pi-alkyl interaction of Lys14. Compound 5 formed hydrogen bonds (including pi-donor) with three shikimate binding residues (Arg116, Arg132, and Asp33). In addition, hydrogen bonds of Gly11, Ser12, Gly13, and Lys14 were assisted by aromaticity modulation (increase in aromaticity upon hydrogen bonding) [54,55]. Compound 6 formed hydrogen bonds via residues Gly13 (d = 2.50 Å) and Glu112 (d = 2.65 Å). It formed three carbon hydrogen bonds through residues Ser15, Lys115, and Asp33. This compound had two intramolecular hydrogen bonds (see S1 Fig). Residue Arg116 formed pi-cation and pi-alkyl interactions, while pi-alkyl interaction was also observed with Met10, Val44, and Lys115. However, compound 6 exhibited two unfavorable donor-donor interactions with residues Gly11 and Gly13. The docked S3P-HpSK complex is shown in Fig 6g (see S1 Fig for 3d interaction). The S3P formed hydrogen bonding with the HpSK via residues Arg57 (d_1_ = 2.17 Å and d_2_ = 2.09 Å), Arg132 (d = 2.17 Å), Arg116 (d = 2.23 Å), Gly80 (d_1_ = 2.13 Å and d_2_ = 2.28 Å), and Asp33 (d_1_ = 2.03 Å and d_2_ = 2.56 Å). Residues Gly79, Pro117, and Met10 each formed a carbon-hydrogen bond with the S3P. Residues Arg57, Arg116, and Lys14 formed attractive charge-charge interactions. Salt bridge (charge-charge) interactions were observed for residues Arg116 and Arg132, while Asp33 displayed two unfavorable negative-negative interactions with the S3P.

Previous experimental evidence indicates that established inhibitors typically target the shikimate-binding site, the ATP-binding site, or both. According to Hsu et al. [20], ligands that established hydrogen bonds with Arg57 and Arg132 within the shikimate-binding site generally exhibited lower inhibition constants. Conversely, repurposed inhibitors such as Cangrelor and Isavuconazonium have been shown to interact with residues outside of this motif during molecular docking [21]. Inhibitor 1 (Fig 2a) interacted with HpSK primarily through hydrogen bonding with Gly11 and Thr78, whereas Inhibitor 2 (Fig 2b) relied on salt bridge formation as the predominant stabilizing force [15].

In the present study, compounds 1, 4, and 5 formed key hydrogen bonds with Arg57 or Arg132, aligning with the interaction patterns observed in high-affinity inhibitors. While compounds 2, 3, and 6 did not rely solely on hydrogen bonding of these specific residues during molecular docking, they exhibited a robust network of hydrogen bonds supplemented by auxiliary non-covalent interactions within the binding pocket. These collective binding profiles suggest that all six candidates possess significant potential as HpSK inhibitors, either by mimicking classical binding modes or through alternative stabilizing interactions.

### 3.2. Molecular dynamics simulation of HpSK

#### 3.2.1. Root mean square deviation (RMSD).

**Fig 7:**
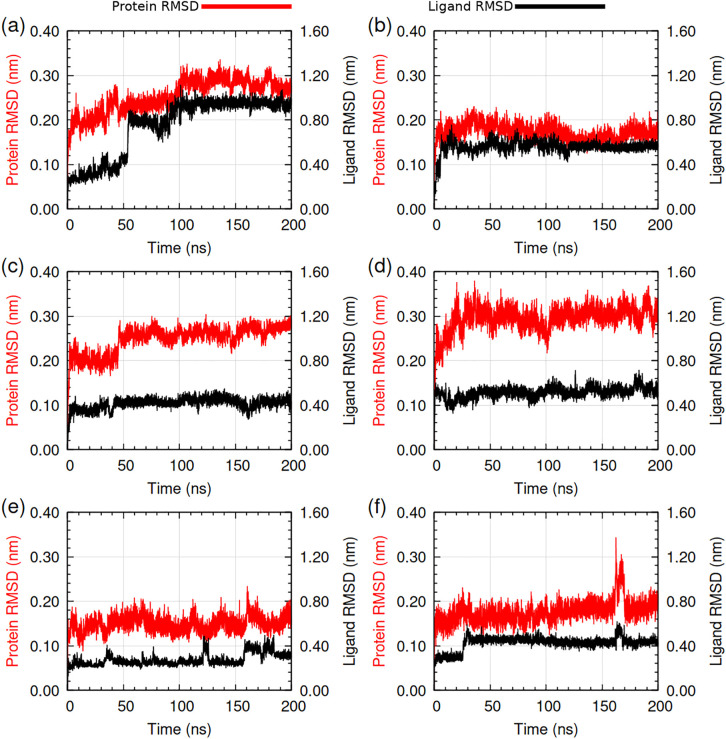
Root mean square deviation (RMSD) of protein backbone and ligand heavy atoms relative to protein backbone. **(a)** HpSK-cpd 1, **(b)** HpSK-cpd 2, **(c)** HpSK-cpd 3, **(d)** HpSK-cpd 4, **(e)** HpSK-cpd 5, and **(f)** HpSK-cpd 6. Protein RMSD is in ‘red’ and ligand RMSD is in ‘black’ color.

Molecular dynamics simulation was performed for the protein-ligand complexes to study the dynamics and stability. Fig 7 (see S2 Fig for RMSD comparison) showed the protein-backbone and ligand heavy atoms RMSD (hereafter stated as ligand RMSD) relative to protein backbone, respectively for compound 1–6 complexes. The protein RMSD of compound 1 was around 0.20 nm at the beginning. It increased gradually and stabilized to around 0.28 nm after 100 ns. Ligand RMSD increased suddenly at 55 ns to 0.80 nm suggesting position or orientation change and stabilized to around 0.94 nm at 100 ns. After 100 ns there was smooth protein and ligand RMSD indicating stabilized protein-ligand complex. During the 200 ns molecular dynamics simulation, compound 2 showed stability of the protein-ligand complex. The protein RMSD remained stable at around 0.15–0.18 nm, and the average ligand RMSD also stabilized to approximately 0.56 nm after 15 ns. The protein RMSD plot of compound 3 showed rise at 45 ns but the average RMSD flattened thereafter to 0.26–0.27 nm. The average ligand RMSD of compound 3 was 0.43–0.48 nm from 40 ns. Overall, compound 3 had smooth ligand and protein RMSD during the 200 ns MDS indicating stable protein-ligand complex.

The protein RMSD of compound 4 stabilized to around 0.30 nm after 20 ns and ligand RMSD stabilized to approximately 0.51 nm. Towards the end of the simulation, minor fluctuations in the protein and ligand RMSD were observed. Overall, the ligand and protein RMSD of compound 4 indicated stabilized protein-ligand complex. The average protein RMSD of compound 5 was around 0.15 nm throughout the simulation with a rise near 160 ns. The ligand RMSD of compound 5 was between 0.25 to 0.38 nm throughout the simulation. The protein and ligand RMSD for compound 5 showed stability during the 200 ns simulation. The protein and ligand RMSD of compound 6 stabilized after 25 ns, with values of 0.17–0.18 nm and 0.42–0.45 nm, respectively. A notable increase in both RMSD values was observed during the 162–167 ns interval. No significant decrease in hydrogen bonds (see Fig 11) was observed during that interval. The RMSD plots indicated a stable protein-ligand complex for compound 6.

**Fig 8:**
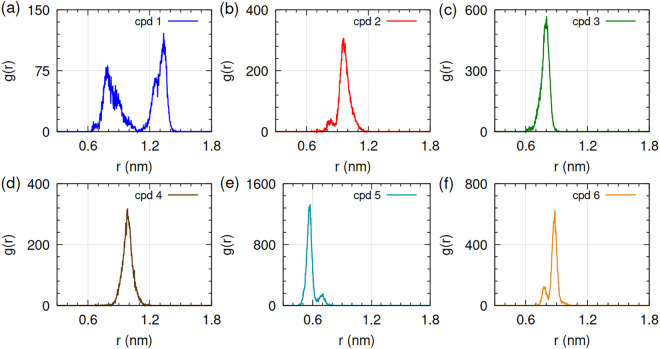
Plot of radial distribution function (g(r)) with distance(r). (a) HpSK-cpd 1, (b) HpSK-cpd 2, (c) HpSK-cpd 3, (d) HpSK-cpd 4, (e) HpSK-cpd 5, and (f) HpSK-cpd 6.

**Fig 9:**
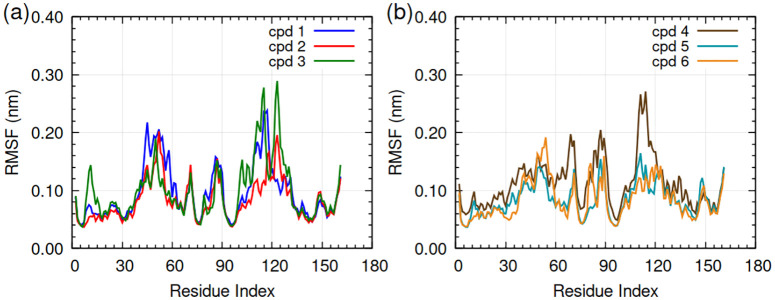
Root mean square fluctuation (RMSF) of residues. **(a)** HpSK-cpd 1-3 and **(b)** HpSK-cpd 4-6. The complexes are indicated by color: HpSK-cpd 1 (blue), HpSK-cpd 2 (red), HpSK-cpd 3 (green), HpSK-cpd 4 (maroon), HpSK-cpd 5 (dark cyan), and HpSK-cpd 6 (golden yellow).

**Fig 10:**
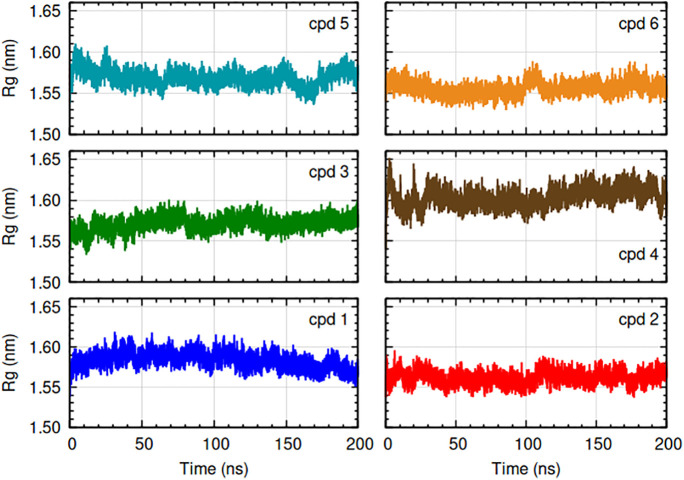
Radius of gyration (Rg) of complexes. The complexes are indicated by color: HpSK-cpd 1 (blue), HpSK-cpd 2 (red), HpSK-cpd 3 (green), HpSK-cpd 4 (maroon), HpSK-cpd 5 (dark cyan), and HpSK-cpd 6 (golden yellow). For clarity, axis labels are shown only on the outer plots but apply to all panels.

**Fig 11:**
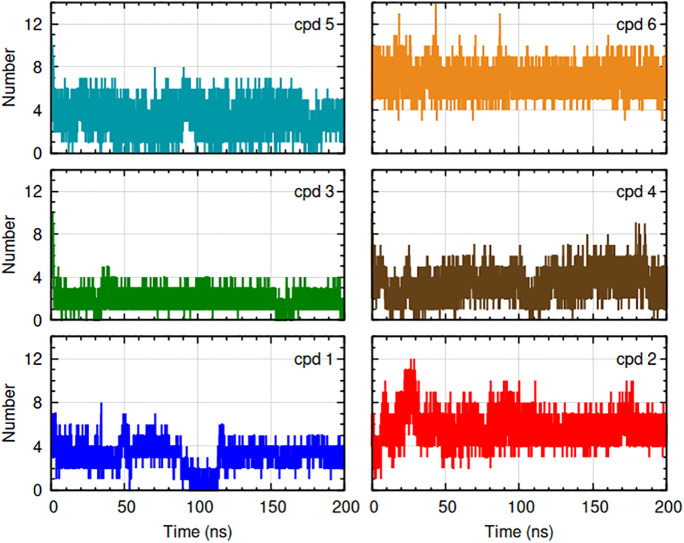
Number of hydrogen bonds between protein and ligands in complexes monitored during 200 ns production runs. The complexes are indicated by color: HpSK-cpd 1 (blue), HpSK-cpd 2 (red), HpSK-cpd 3 (green), HpSK-cpd 4 (maroon), HpSK-cpd 5 (dark cyan), and HpSK-cpd 6 (golden yellow). For clarity, axis labels are shown only on the outer plots but apply to all panels.

Compound 4 showed a protein RMSD of approximately 0.30 nm, which, despite being the group’s highest value, is within acceptable limits [58]. The trajectory reached a clear structural equilibrium after the initial 20 ns and remained stable thereafter. The reliability of this complex was further supported by a consistent ligand RMSD and a radial distribution function (RDF) plot (Fig 8d) featuring a single, sharp peak, indicating a well-defined interaction distance. Based on this observed structural convergence and its favorable hydrogen-bonding profile with key residues, compound 4 was justified for inclusion in the final list of potential leads.

#### 3.2.2. Radial distribution function (RDF) analysis.

The radial distribution function g(r), gives the probability of finding the positions of an atom-pair within a specified range of radial distance during MD simulations [59]. The RDFs (Fig 8) of center of mass of ligand with respect to center of mass of protein were extracted from MD trajectory. For compound 1, the presence of two distinct peaks in the radial distribution function (RDF) indicated that the ligand occupied two separate radial distances over the course of the simulation. This observation is consistent with the ligand RMSD transition observed at approximately 52 ns, suggesting a discrete conformational reorientation or a positional shift of the ligand within the binding pocket during the trajectory. Remaining compounds 2, 3, 4, 5, and 6 had one sharp peak, which pointed towards their localization around the protein. A similar procedure was used to analyze protein-ligand complexes in literature [60]. RDF analysis revealed a relatively constant interaction distance between compounds and receptor protein except that of compound 1. These observations supported findings from RMSD and ligand snapshots analysis (see S1 Fig).

#### 3.2.3. Root mean square fluctuation (RMSF).

Fig 9a and b showed the RMSF plot of HpSK-compound 1–6 complexes. Enhanced conformational flexibility was observed within the LID region (residues 109–123) and the region spanning residues 45–52 of the HpSK-Compound 1 complex, which contributed to a higher overall protein RMSD. The peaks in the RMSF curve of the HpSK-compound 1 complex were observed at residues 45 (0.22 nm), 52 (0.21 nm), 115 (0.24 nm), 116 (0.23 nm), and 117 (0.24 nm); the remaining residues had fluctuation below 0.20 nm. The peaks in the RMSF curve of HpSK-compound 3 complex corresponded to residue numbers 114 (0.26 nm), 115 (0.28 nm), 122 (0.26 nm), and 123 (0.29 nm). The greater flexibility of LID region in HpSK-compound 3 complex implied it had weaker interaction with the LID region residues during the course of simulation.

The RMSF of HpSK-compound 4 complex showed that residue numbers 110–115, within the LID region, had fluctuation of approximately 0.23–0.27 nm, the remaining residues had fluctuation below 0.21 nm. The complexes of compounds 2, 5, and 6 demonstrated a consistent RMSF below 0.21 nm for all residues over the 200 ns MD simulation period. The low RMSF for LID region residues meant the complexes of compounds 2, 5, and 6 were in constant binding with those residues or overall interactions with the protein were stronger during 200 ns simulation. Overall, lower RMSF for the complexes of compounds 2, 5, and 6 resulted in lower protein RMSD for them compared to the complexes of compounds 1, 3, and 4.

#### 3.2.4. Radius of gyration (Rg).

Fig 10 showed radius of gyration (Rg) plots of HpSK-compound 1–6 complexes. The radius of gyration of compound 1 was around 1.58 nm for most of the time and around 1.57 nm towards the end of simulation. There were no major contractions or expansions throughout the simulation indicating compact and stable protein structure. The radius of gyration of compound 2 (approximately 1.56 nm) showed compact and stable protein structure during the 200 ns MD simulation. The radius of gyration of compound 3 stabilized to around 1.57 nm without major expansion and contraction indicating compact protein during the simulation. The radius of gyration of compound 4 stabilized to around 1.60–1.61 nm. Absence of major expansion and compaction during the 200 ns simulation suggested a stable protein-ligand complex. The radius of gyration of compound 5 was stable with an average of 1.57 nm throughout the course of simulation. The Rg plot showed a compact and stable protein structure. The radius of gyration of compound 6 stabilized to around 1.55–1.56 nm, with a rise during the 100–110 ns period.

#### 3.2.5. Hydrogen bond analysis.

Hydrogen bond counts between the protein and ligand in their complex is shown in Fig 11. The average hydrogen bond count for compound 1 was 2–4, except during the 90–113 ns period where it was 0–2. Compound 2 had 4–7 hydrogen bonds; compound 3 had 1–3 hydrogen bonds; and compound 6 had 5–8 hydrogen bonds. Nearly consistent hydrogen bonds for 2, 3, and 6 hinted toward a smooth RMSD curve for those compounds. Compound 4 and 5 had 2–5 hydrogen bonds throughout the simulation. Presence of notable frames with lower (0 and 1) as well as higher (6, 7 and 8) hydrogen bonds in compound 5 possibly contributed to spikes in its protein RMSD.

## 4. Conclusion

In this study, natural products from the North African Natural Product Database (NANPDB) and East African Natural Product Database (EANPDB) were evaluated as potential inhibitors of *H. pylori* shikimate kinase. Molecular docking identified six lead compounds exhibiting superior binding energies and interaction profiles compared to the reference ligand, shikimate 3-phosphate. Beyond conventional hydrogen bonding, the protein-ligand complexes for Compounds 2 and 5 were uniquely stabilized by aromaticity-modulated hydrogen bonds, while compound 2 was further bolstered by a rare anion-pi-cation ternary interaction.

These docking results were rigorously substantiated by 200 ns molecular dynamics (MD) simulations, which confirmed the long-term structural stability of the complexes. Throughout the trajectory, compounds 2, 4, 5, and 6 maintained a high density of hydrogen bonds. Notably, Compound 5 demonstrated the most robust performance, forming hydrogen-bonding interactions with key residues Arg57, Arg132, and Asp33 within the docked complex. The convergence of favorable docking scores, unique stabilizing motifs, and MD-derived stability profiles highlights compound 5 as the most promising scaffold for subsequent experimental validation. These identified natural products provide a compelling starting point for the development of novel antibiotics targeting the shikimate pathway in *H. pylori*.

## Supporting information

S1 Fig 3d interaction diagram of complexes. (a) HpSK-cpd 6 and (b) HpSK-S3P.
(TIFF)

S2 Fig Plot of protein RMSD and ligand RMSD of complexes. (a) Protein RMSD and (b) Ligand RMSD. The complexes are indicated by color: HpSK-cpd 1 (blue), HpSK-cpd 2 (red), HpSK-cpd 3 (green), HpSK-cpd 4 (maroon), HpSK-cpd 5 (dark cyan), and HpSK-cpd 6 (golden yellow).
(TIFF)

S1 File Binding site snapshots analysis during molecular dynamics simulation. (DOC)
