# Supplementary figures and images for: Computational investigation of African natural products as Helicobacter pylori shikimate kinase inhibitors
Source: PLoS One. 2026 Apr 20;21(4):e0346899. doi: 10.1371/journal.pone.0346899 (PMC13094947; doi:10.1371/journal.pone.0346899)

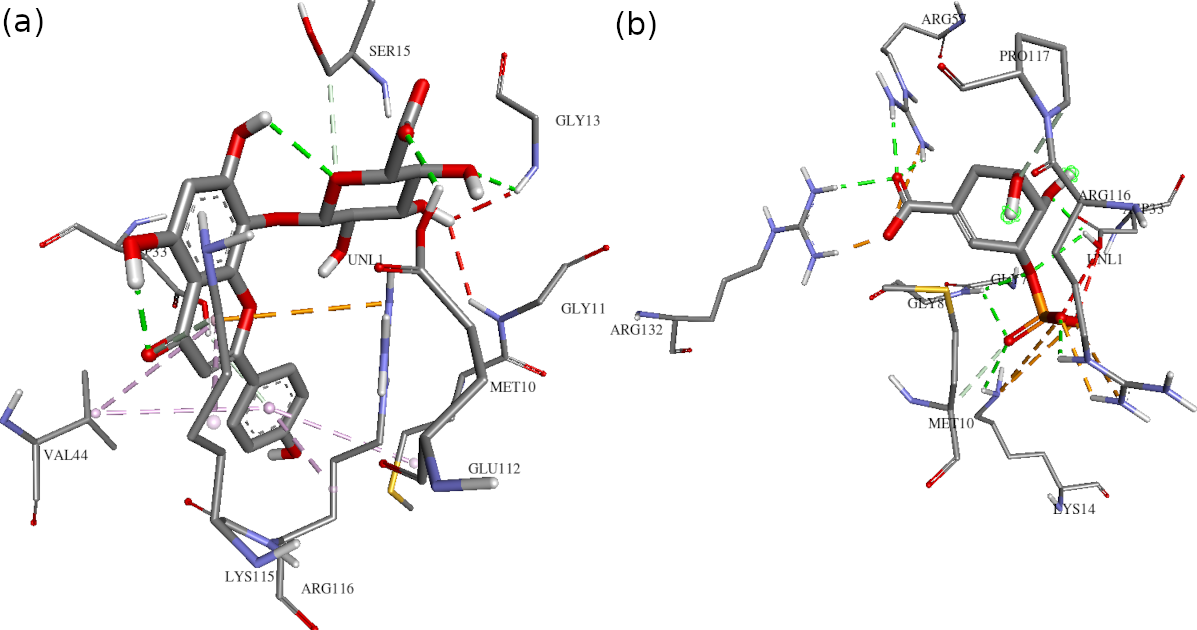

Supplement: S1 Fig — (a) HpSK-cpd 6 and (b) HpSK-S3P. (TIFF) [file pone.0346899.s001.tiff]

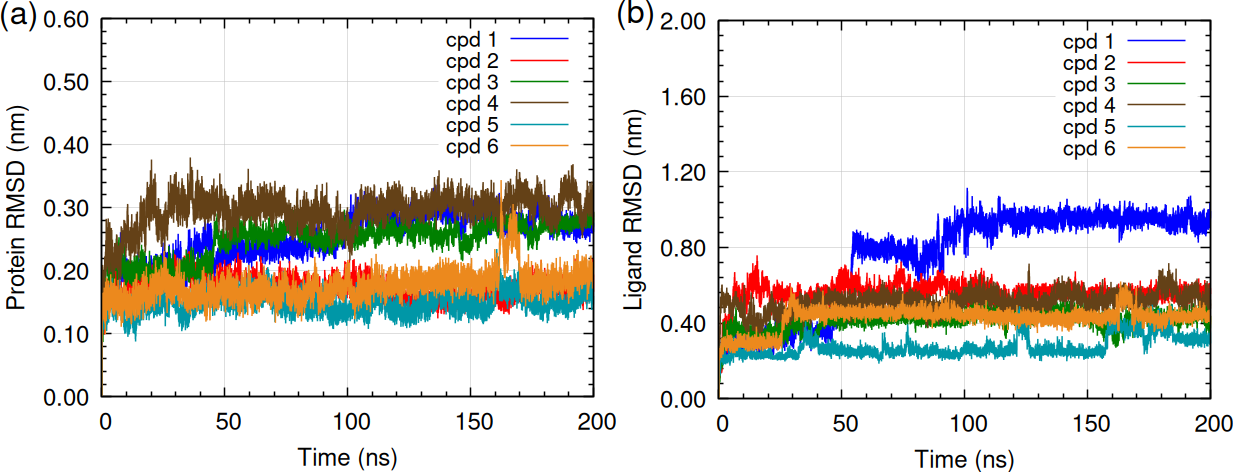

Supplement: S2 Fig — (a) Protein RMSD and (b) Ligand RMSD. The complexes are indicated by color: HpSK-cpd 1 (blue), HpSK-cpd 2 (red), HpSK-cpd 3 (green), HpSK-cpd 4 (maroon), HpSK-cpd 5 (dark cyan), and HpSK-cpd 6 (golden yellow). (TIFF) [file pone.0346899.s002.tiff]
