# Supplementary material for: Computational investigation of African natural products as Helicobacter pylori shikimate kinase inhibitors
Source: PLoS One. 2026 Apr 20;21(4):e0346899. doi: 10.1371/journal.pone.0346899 (PMC13094947; doi:10.1371/journal.pone.0346899)
Supplement: S1 File — (DOC) [file pone.0346899.s003.doc]

Computational Investigation of African Natural Products as *Helicobacter pylori* Shikimate Kinase Inhibitors

**Binding site snapshots analysis during molecular dynamics simulation**

**S1 Table. Ligand snapshots of compounds during 200 ns molecular dynamics simulation.** Ligand is shown in line model and protein in cartoon representation.


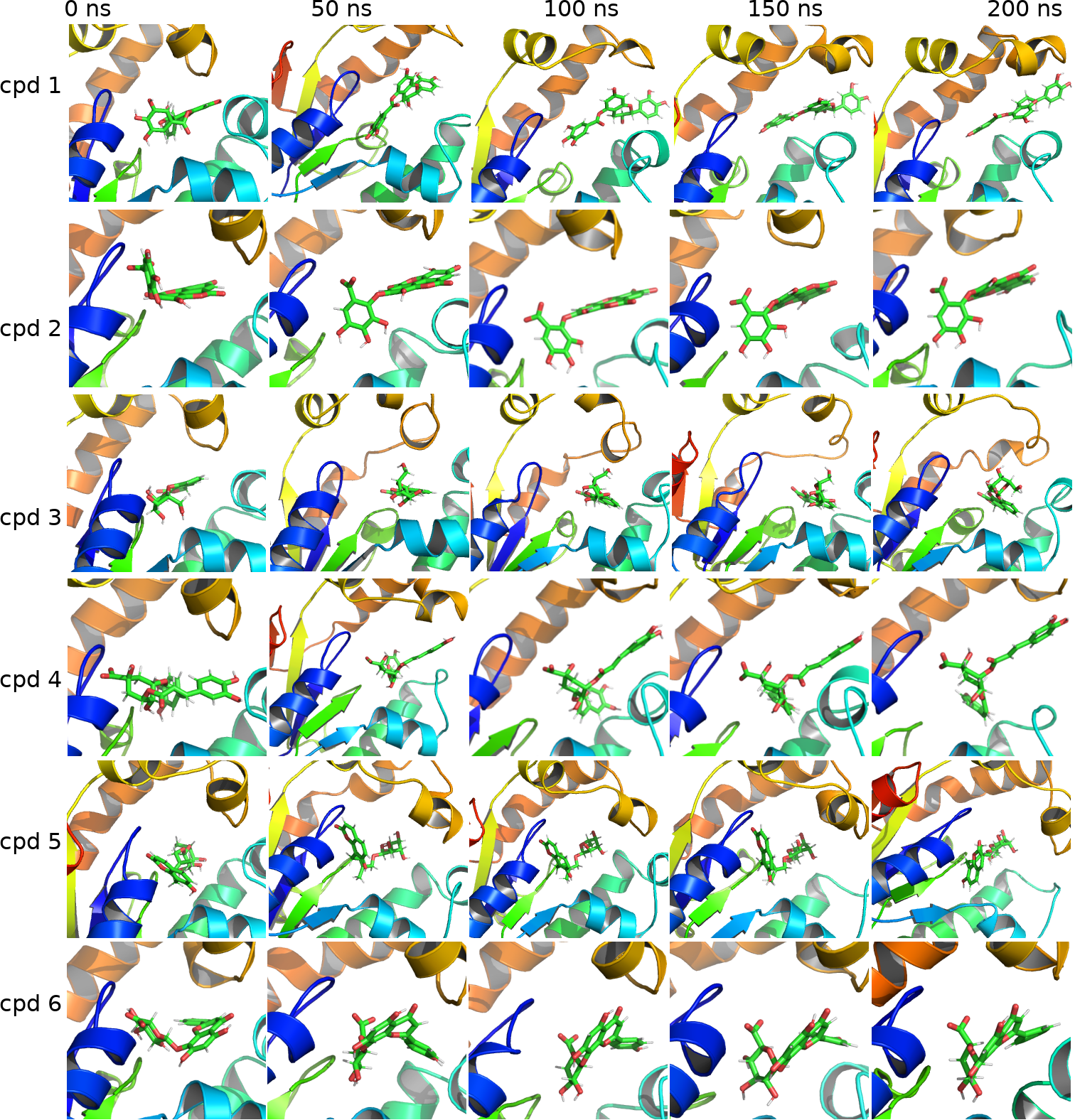


Ligand snapshots were extracted at 0, 50, 100, 150 , and 200 ns time of MDS to study the position and orientation of ligands as shown in S1 Table. Compound 1 had nearly the same location in 0 and 50 ns with significant change in orientation. Then, it adopted a new position after 50 ns. Snapshots at 100, 150, and 200 ns showed ligand was in the same position with notable change in orientation. Ligand RMSD of compound 1 also showed sudden rise around 50 ns, consistent with this observation. Compound 2 snapshots at 0, 50, 100, 150, and 200 ns showed that ligand remained in nearly the same position. The orientations were slightly different after the initial 0 ns frame, indicating minor rotational flexibility throughout the simulation. Due to absence of significant conformational and translational changes, its ligand RMSD was smooth with minimal spikes from 15 ns to end of the simulation. Compound 3 appeared to change position at 50 ns, but snapshots at 100, 150, and 200 ns showed that ligand remained in nearly the same position with changed orientation, which demonstrated its stability. **Owing to the absence of significant conformational and translational changes, the ligand RMSD remained stable, exhibiting minimal fluctuations throughout the simulation.** Compound 4 had nearly the same position at 50, 100, 150, and 200 ns with notable change in orientation except that of 0 ns. Compound 5 had the minimal delocalization with notable change in orientation throughout the 0, 150, 100, 150, and 200 ns snapshots. Due to negligible translational motion of ligands, compound 5 had lower ligand and protein RMSD compared to compounds 1- 4. Compound 6 snapshots at 0, 50, 100, 150, and 200 ns showed minimal delocalization with notable orientation change.
